# Molecular Cloning and Induced Expression of Six Small Heat Shock Proteins Mediating Cold-Hardiness in *Harmonia axyridis* (Coleoptera: Coccinellidae)

**DOI:** 10.3389/fphys.2017.00060

**Published:** 2017-02-09

**Authors:** Hui-Juan Wang, Zuo-Kun Shi, Qi-Da Shen, Cai-Di Xu, Bing Wang, Zhao-Jun Meng, Shi-Gui Wang, Bin Tang, Su Wang

**Affiliations:** ^1^College of Life and Environmental Sciences, Hangzhou Normal UniversityHangzhou, China; ^2^Institute of Plant and Environment Protection, Beijing Academy of Agriculture and Forestry SciencesBeijing, China; ^3^College of Forestry, Northeast Forestry UniversityHarbin, China

**Keywords:** *Harmonia axyridis*, super cooling point, freezing point, cold-hardiness, small heat shock protein, qRT-PCR, gene expression

## Abstract

The main function of small heat shock proteins (sHSPs) as molecular chaperones is to protect proteins from denaturation under adverse conditions. Molecular and physiological data were used to examine the sHSPs underlying cold-hardiness in *Harmonia axyridis*. Complementary DNA sequences were obtained for six *H. axyridis* sHSPs based on its transcriptome, and the expression of the genes coding for these sHSPs was evaluated by quantitative real-time PCR (qRT-PCR) in several developmental stages, under short-term cooling or heating conditions, and in black and yellow females of experimental and overwintering populations under low-temperature storage. In addition, we measured water content and the super cooling and freezing points (SCP and FP, respectively) of *H. axyridis* individuals from experimental and overwintering populations. The average water content was not significantly different between adults of both populations, but the SCP and FP of the overwintering population were significantly lower than that of the experimental population. Overall, the six sHSPs genes showed different expression patterns among developmental stages. In the short-term cooling treatment, *Hsp16.25* and *Hsp21.00* expressions first increased and then decreased, while *Hsp10.87* and *Hsp21.56* expressions increased during the entire process. Under short-term heating, the expressions of *Hsp21.00, Hsp21.62, Hsp10.87*, and *Hsp16.25* showed an increasing trend, whereas *Hsp36.77 first* decreased and then increased. Under low-temperature storage conditions, the expression of *Hsp36.77* decreased, while the expressions of *Hsp21.00* and *Hsp21.62* were higher than that of the control group in the experimental population. The expression of *Hsp36.77 first* increased and then decreased, whereas *Hsp21.56* expression was always higher than that of the control group in the overwintering population. Thus, differences in sHSPs gene expression were correlated with the *H. axyridis* forms, suggesting that the mechanism of cold resistance might differ among them. Although, *Hsp36.77, Hsp16.25, Hsp21.00*, and *Hsp21.62* regulated cold- hardiness, the only significant differences between overwintering and experimental populations were found for *Hsp16.25* and *Hsp21.00*.

## Introduction

*Harmonia axyridis* (Coleoptera: Coccinellidae) has strong predation ability on aphids, spider mites, mealy bugs, and other important pests, thus being an important natural enemy (Koch, 2003). Due to its use in agricultural productions worldwide, it is now a cosmopolitan invasive species causing negative ecological impacts (Koch, 2003; Wang et al., 2015). The survival of *H. axyridis* winter adults is an important factor affecting population's offspring (Zhao et al., 2008). Therefore, several studies on cold hardiness and overwintering strategies, as well as on the relationship between seasonal phenotypic plasticity and cold temperatures, have been developed in this species (Koch et al., 2004; Labrie et al., 2008; Wang et al., 2009; Berkvens et al., 2010; Michie et al., 2011; Ruan et al., 2012).

Under natural conditions, temperature greatly influences insect growth and development, their basic behavior, and evolutionary path (Lee and Denlinger, 1991). Lee (1989) defined cold tolerance as the survival ability of a living creature exposed to low temperature, for a long or short time, according to seasonal environmental changes, genetic factors, nutritional status, and the length of exposure to low temperature. Insects have evolved a variety of cold-resistance measures (Jing and Kang, 2002), mainly including ecological (or behavior) and physiological aspects. Whereas, the former include migrating or hiding to avoid the damages caused by low temperature, the latter include regulating the body's metabolic mechanisms and the accumulation of cold resistant compounds such as glycerol, trehalose, and polyol, among others (Watanabe, 2002; Guo et al., 2006; Gagnon et al., 2013). Many studies on insects' cold tolerance used the super cooling point (SCP) as an important indicator of the strength of cold tolerance (Nedved et al., 1998; Renault et al., 2002; Koch et al., 2004; Colinet et al., 2006). In natural conditions, insects experience a process of cold acclimation before winter that increases cold tolerance, helping to withstand the low temperature environment (Leather et al., 1993). Experiments showed that the cold tolerance of *H. axyridis* overwintering populations was higher than that of summer populations, due, to some extent, to winter acclimation (Zhao et al., 2010). Studies also indicated that insects moderating cold acclimation before low temperature stress have higher survival rate, lower lethal temperature, prolonged half-lethal time, and decreased SCP (Fields et al., 1998; Renault et al., 2004; Ma et al., 2006; Wang et al., 2006).

Heat shock proteins (HSPs) are highly conserved amino acid sequences, which are widely found in microorganisms, plants, and animals. They function as molecular chaperones to protect proteins from being denatured in high temperature stress (Montfort et al., 2001; Sun and MacRae, 2005), but also protect proteins under cold, drought, oxidation, hypertonic stress, UV, and heavy metals exposure (Dasgupta et al., 1992; Waters et al., 2008; Meijering et al., 2012; Senf, 2013), or under high population density (Wang et al., 2007). The classification of HSPs into five families, namely HSP100, HSP90, HSP70, HSP60, and small HSPs (sHSPs; Carper et al., 1987; Kim et al., 1998), is mainly based on their molecular weight and homologs relationships. With the exception of sHSPs, which are more diverse than the other HSPs, all these families are conserved among organisms (Li et al., 2009). As a family of molecular chaperones, small heat shock proteins (sHSPs) are characterized by a low subunit molecular mass (12–43 kDa), a conserved a-crystallin domain, and for forming large oligomers (Zhang et al., 2015). Small HSPs are conserved across species and play an important role in various developmental, biotic, and abiotic stresses (Bakthisaran et al., 2015; Pandey et al., 2015). The number of genes encoding sHSPs varies greatly among organisms, from as few as one to as many as 19 (Haslbeck et al., 2005). Small HSPs are widely distributed across tissues and play an important role in cell survival under stress conditions (Bakthisaran et al., 2015). The reversible dissociation of the homo-oligomers formed by sHSPs has been reported as important for their enhanced chaperone activity (Haslbeck et al., 1999, 2008).

Since their first description in fruit flies, great advances in the study of HSPs revealed their high conservation throughout evolution, suggesting they might have a vital role in protecting cells from injury (Luo et al., 2007). As molecular chaperones, HSPs participate in protein folding and degradation and in the transport of intracellular material. When stimulated, HSPs can promote protein folding, recovering their original spatial structure and biological activity. In the absence of external stimulation, HSPs can also promote peptide chain folding and ligation. Being a survival gene, *Hsp70* can be rapidly expressed to build up protection against several cellular stresses, including elevated temperatures, mechanical damage, hypoxia, lowered pH, and reactive oxygen species generation (Yong et al., 2015). In addition, HSP90 has emerged as a major pharmaceutical target in cancer therapy, and it has been proven responsible for indirectly inducing multiple pathways leading to angiogenesis and metastasis in cancer (Kim et al., 2015; Sharma et al., 2016). Heat shock proteins are ubiquitously present in cells and can modulate several cellular functions; inhibition of *Hsp27* and *Hsp40* potentiates 5-fluorouracil and carboplatin mediated cell killing in hepatoma cells (Sharma et al., 2009). As different classes of HSPs play several roles in governing proper protein assembly, folding, and translocation (Hightower, 1991; Hartl, 1996), the regulation of their synthesis establishes a unique defense system to maintain cellular protein homeostasis and to ensure cell survival (Hartl, 1996).

The elytra of *H. axyridis* adults have a rich color, usually black (melanic) or yellow (non-melanic) stained with red, orange, or black dots (Dobzhansky, 1933), which results from a series of expressed alleles (Tan and Li, 1934; Tan, 1946). Most of these alleles are rare in *H. axyridis* populations, with a combined frequency of <1%, except for the four major alleles after which the four major forms of *H. axyridis* are named—f. *conspicua*, f. *spectabilis*, f. *axyridis*, and f. *succinea* (Michie et al., 2010). Because stain ratios differ among areas and seasons within the same area (Heimpel and Lundgren, 2000; Seo and Youn, 2000; Wang et al., 2009; Michie et al., 2011), environmental factors might lead to such elytral diversity (Tang et al., 2012). In the north of China, *H. axyridis* melanic and non-melanic types vary seasonally: in the summer adults are mainly black and the number of yellow type adults significantly increases in the autumn (Wang et al., 2009); in winter, the proportion of yellow type *H. axyridis* was significantly higher than that of black adults. To survive through the cold winter, *H. axyridis* individuals move to concealed and sheltered locations where they aggregate, creating a protective microclimate in which insects experience less extreme temperatures than in the surrounding areas (Berkvens et al., 2010; Durieux et al., 2015). In Northeast China, adults aggregate in some fixed locations where they overwinter (Wang et al., 2011). Although, pre-wintering and overwintering *H. axyridis* populations increase cold tolerance and compounds' storage during extended periods at low temperature (Ruan et al., 2012), the changes occurring in their metabolism to achieve cold tolerance in winter have rarely been reported. Therefore, the water content, SCP, and freezing point (FP) of experimental and overwintering *H. axyridis* populations were obtained to analyze their cold tolerance. Six sHSPs were cloned and their differential expression at several developmental stages, short-term cooling temperature stress, and low-temperature storage conditions was determined, to study the potential molecular mechanisms of cold resistance in *H. axyridis*.

## Materials and methods

### Insects

Experimental populations of *H. axyridis* were collected from the Lab of Natural Enemy Research, Beijing Academy of Agriculture and Forestry Science, and reared and maintained in our laboratory over a 3-year period. Non-melanic and melanic populations were separated, maintained at 25°C, 70% relative humidity, and 16:8 h (light:dark) photoperiod, and fed *Aphis medicaginis*. At each molt, developmental stages were synchronized by collecting new larvae, pupae, or adults. Overwintering populations were collected from Heilongjiang province, Northeast of China, in 2015. After *H. axyridis* elytrum-staining stabilized, adults were divided into black (melanic) and yellow (non-melanic) types, according to their elytra background.

### Water content, SCP, and FP determination

The wet and dry weights of each insect within each group (*n* = 15) were determined, and the water content of each insect's body was calculated as the difference between wet and dry weight (weight of body water) divided by the wet weight.

The thermocouple method was used to measure SCP and FP, by determining latent heat release (Ju and Du, 2002). As the insect's body temperature cools below the SCP, body fluids begin to freeze spontaneously. A temperature probe was fixed on the back of each *H. axyridis* individual and connected to a computer, which automatically recorded and processed body temperature variations. *H. axyridis* were placed in a refrigerator and their body temperature declined with decreasing temperature down to a point where it stopped decreasing and began to rise. This temperature is the SCP and that at which body temperature begins to decline again is the FP (Liu et al., 2005). A cooling curve was drawn, and the SCP was read from it.

### Cloning six sHSPs genes

In our previous study (Tang et al., 2017), two *H. axyridis* groups were exposed to normal and low temperature conditions (5°C) for 2 h. These groups were named HaRT_Trans and HaCS_Trans, respectively, and their transcriptome sequencing and analysis revealed partial sequences of six *sHsps* genes. In the present study, total RNA was isolated from *H. axyridis* using TRIzol® reagent (Invitrogen, Shanghai, China), and first-strand cDNA synthesis was carried out using a PrimeScript RT® with gDNA Eraser kit (TaKaRa, Dalian, China). Based on the six sHSPs found in the previous study, specific primers were designed and used to obtain full length cDNA sequences by Rapid Amplification of cDNA Ends (RACE) technology, together with a SMART™ kit (TaKaRa) and following the manufacturer's protocol. The resulting PCR products were separated by electrophoresis on 1.0% agarose gels, and the cDNA fragments of interest were purified using a DNA gel extraction kit (OMEGA, Hangzhou, China). Purified DNA was ligated into a pMD18-T vector (TaKaRa) and sequenced using the Sanger method.

### Sequence and phylogenetic analysis

Nucleic acid sequences of *H. axyridis* sHSPs were queried for similar sequences on the National Center for Biotechnology Information (NCBI). Multiple sHSPs sequences, belonging to *H. axyridis* and other insects, were aligned using ClustalW (Julie et al., 2002), and a neighbor-joining (NJ) phylogenetic tree was constructed in Mega 7.0 software and evaluated using 1000 bootstrap replications.

### Expression of sHSPs genes in different developmental stages

The experimental population was used in this trial. Total RNA was extracted from larvae, pre-pupae, pupae, and adults (four individuals from each developmental stage; collected 1–3 days after molting or 2 h after eclosion) as described in the previous section. After cDNA synthesis (as described in the previous section), the relative expression of sHSPs genes was detected by quantitative real-time PCR (qRT-PCR). Primers used in qRT-PCR were designed based on the conserved regions of sHSPs genes from differently colored *H. axyridis* (Table 1). Details of the qRT-PCR are given below, in the section “Quantitative RT-PCR.”

**Table 1:** **Primer sequences used in quantitative real-time PCR**.

**Primer name**	**Nucleotide sequences (5′–3′)**
Ha-rp49-QF	GCGATCGCTATGGAAAACTC
Ha-rp49-QR	TACGATTTTGCATCAACAGT
HaHsp36.77-F	TTCTTCACGGCTGTCTT
HaHsp36.77-R	AATCACTGCCTTCCCTC
HaHsp16.25-F	GACCCTCAACATACCAGA
HaHsp16.25-R	TGATGCTTACCCTTTACTTC
HaHsp21.00-F	CACCATAGAAGGCAAGCA
HaHsp21.00-R	AGACAATCTCGATTCCACC
HaHsp21.62-F	TCTTCTGGACCAACATTTC
HaHsp21.62-R	GTGGCTTTAACGGTGATT
HaHsp10.87-F	TGCCCTTGTTGGATAGA
HaHsp10.87-R	TGTTGCCTTCAGGACTT
HaHsp21.56-F	AGGAGCATGGTGGACTG
HaHsp21.56-R	ACTTCACTTTCTGGCAATC

### Short-term cooling and heating

The experimental population was used in this trial. Lee and Denlinger (1991) found that rapid cooling contributed to enhance cold-hardiness in arthropods. As the coming of winter or spring is a process of gradual cooling or warming, we designed a series of experiments using different temperatures (25, 15, 10, 5, 0, and −5°C) to measure sHSPs responses to temperature transitions. *H. axyridis* were placed in rapidly changing temperature environments. In treatment (i), hundreds of individuals were placed in plastic tubes sealed with a sponge (10 individuals per tube) and maintained at 25°C for 2 h; tubes were then rapidly cooled to 15°C, kept at this temperature for 2 h, rapidly cooled to 10°C, kept at this temperature for 2 h, and finally rapidly cooled to −5°C. Treatment (ii) involved a similar procedure, but the starting temperature was −5°C and individuals were heated to 25°C. As a control, 100 adults were maintained at 25°C without any cold stimulation before treatment (ii) (Shi et al., 2016). We randomly sampled three pieces of abdominal tissue from experimental insects at every changing temperature point. The above treatments were repeated three times. Total RNA isolation and cDNA synthesis were performed as described in the “Cloning six sHSPs genes” section, and details of the qRT-PCR are given below, in the “Quantitative RT-PCR” section.

### Low temperature storage

Black and yellow females from the experimental and overwintering *H. axyridis* populations stored at low temperature (5°C) were sampled at 0, 5, 10, 15, and 20 days after storage. At each time point, 5–10 individuals were removed and analyzed. The experiment was repeated three times.

### Quantitative RT-PCR

Total RNA was isolated from *H. axyridis* adults after cold induction, and 1 μg total RNA was used to synthesize first-strand cDNA, as described in the “Cloning six sHSPs genes” section. The expression levels of the six sHSPs genes obtained from cloning, namely *Hsp36.77, Hsp16.25, Hsp21.00, Hsp21.62, Hsp10.87*, and *Hsp21.56*, were estimated by qRT-PCR in a CFX96™ system, using the SsoFast™ EvaGreen® Supermix (both Bio-Rad Laboratories, Hercules, CA, USA). The qRT-PCR was performed in a 20 μl total reaction volume containing 1 μl cDNA template, 1 μl (10 μmol/μl) each primer, 7 μl RNase-free and DNase-free water, and 10 μl SsoFast™ EvaGreen® Supermix. Gene expression data was normalized using the housekeeping gene *Harp49* (*H. axyridis* ribosomal protein 49 gene, AB552923) as the internal control (Shi et al., 2016), which was amplified using the primers Harp49-qF (5′-GCGATCGCTATGGAAAACTC-3′) and Harp49-qR (5′-TACGATTTTGCATCAACAGT-3′). Primers for the six sHSPs genes of *H. axyridis* were designed to target their unique regions; the annealing temperature of each primer pair is shown in Table 1. Target amplification efficiency was identical to that of the reference amplification at each annealing temperature. The cycling parameters were 94°C for 5 min (initial denaturation), followed by 40 cycles at 94°C for 15 s, 59°C for 30 s, and 65°C for 30 s; the fluorescence signal was collected at 68°C. Expression was quantified using the ΔΔCt relative method (Shi et al., 2016).

### Statistical analysis

Data normality and variance homogeneity was evaluated based on three replicates at each temperature point. A multiple factorial three-way analyses of variance (ANOVA) was used to evaluated differences in water content, SCP, and FP among groups, considering color, treatments, and sex as independent factors. The expressions of sHSPs genes during cooling or heating processes were analyzed in IBM SPSS 22 (IBM Corporation, Armonk, NY, USA), and multiple comparisons of means were conducted using Tukey's test. Differences between means were considered significant when *P* < 0.05, and extremely significant when *P* < 0.01.

## Results

### Water content, SCP, and FP

**Figure 1:**
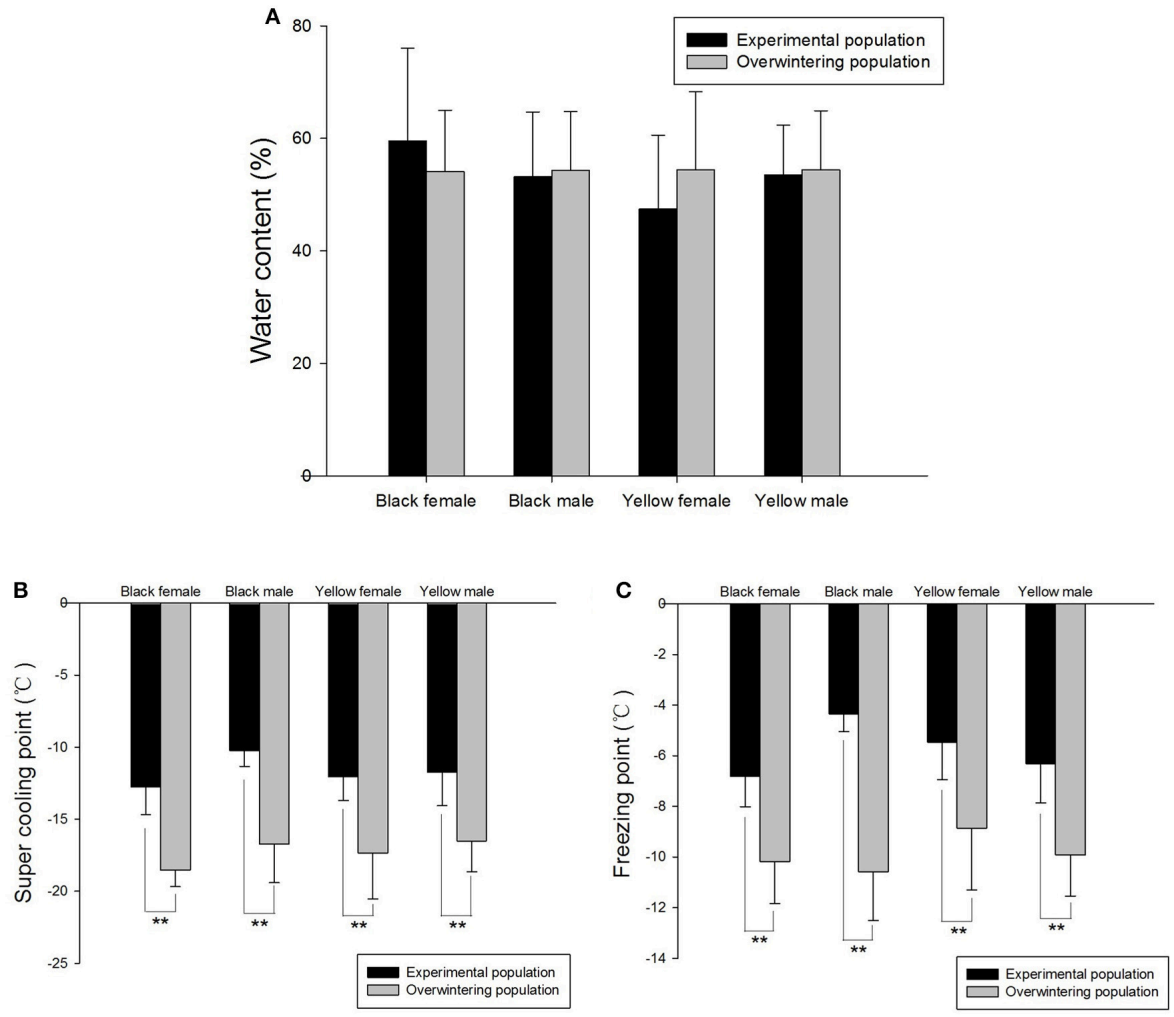
**Water content (A)**, super cooling point **(B)**, and freezing point **(C)** of *Harmonia axyridis* in different populations. The overwintering population was obtained from Northeast China and the experimental population from Hangzhou. Bars with double asterisks indicate significant differences (*P* < 0.01).

The average water contents of overwintering and experimental populations were 54.27 and 53.42%, respectively. There were no obvious differences between the same forms of *H. axyridis* adults belonging to overwintering or experimental populations (Figure 1A). The water content of black adults from the experimental population (56.34%) was slighter higher than that of yellow adults (50.50%), but no obvious differences between adult forms were detected in the overwintering population. There was no significant difference in water content between sexes in the overwintering or experimental populations.

The SCP and FP of the overwintering population were −17.28 and −9.89°C, respectively, whereas those of the experimental population were −11.70 and −5.74°C, respectively. For the same forms of *H. axyridis* adults, SCP and FP were significantly lower in the overwintering than in the experimental population (Figures 1B,C). Three-way ANOVA results showed there was no significant interaction between color, sex, and treatment (Table 2). Females' SCP was significantly lower than that of males within same color *H. axyridis* adults (Figure 1B). There was a small gap in the FP and SCP of differently colored insects within the overwintering population, with the SCP and FP of black adults being slightly lower than that of yellow adults, although this difference was not significant (Table 2).

**Table 2:** **Analysis of variance table realized by GLMM**.

**Source**	**Sum Sq**.	**df**	**Mean Sq**.	***F***	**Sig**.
Color	0.787	1	0.787	0.174	0.677
Treats	932.084	1	932.084	206.109	0
Sex	55.733	1	55.733	12.324	0.001
Color^*^treats	9.163	1	9.163	2.026	0.157
Color^*^sex	18.961	1	18.961	4.193	0.043
Treats^*^sex	0.08	1	0.08	0.018	0.894
Color^*^treats^*^sex	2.682	1	2.682	0.593	0.443
Error	506.496	112	4.522		
Total	26718.881	120			

*R^2^ = 0.668 (Corrected R^2^ = 0.647)*.

### Phylogenetic analysis of sHSPs

**Figure 2:**
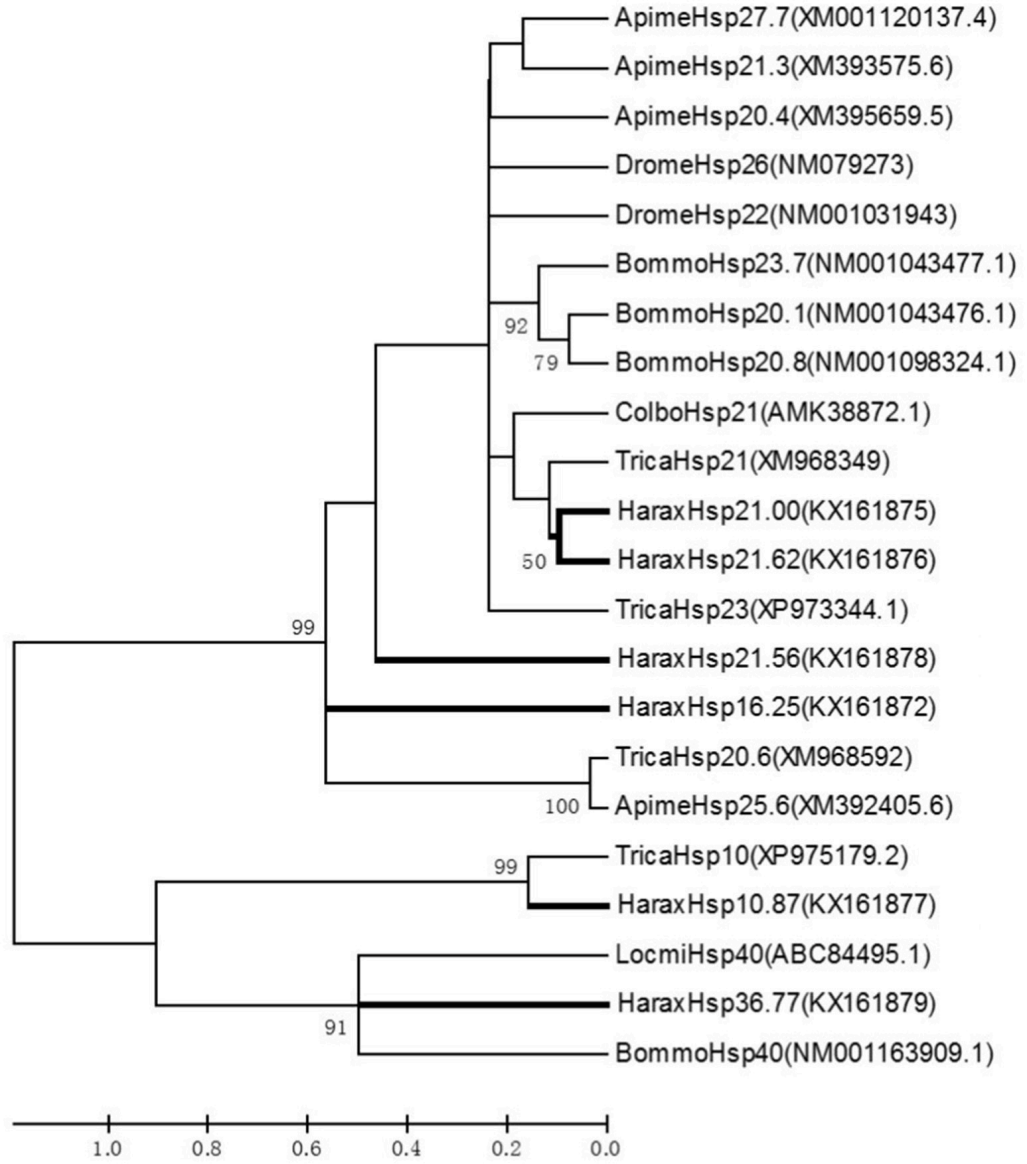
**Phylogenetic analysis of sHSPs in insects**. Phylogenetic tree constructed by the neighbor-joining method. Bootstrap values for 1000 replications are indicated at each node. Scale bar = 0.2 PAM. Harax, *Harmonia axyridis*; Apime, *Apis mellifera*; Bommo, *Bombyx mori*; Colbo, *Colaphellus bowringi*; Drome, *Drosophila melanogaster*; Locmi, *Locusta migratoria*; Trica, *Tribolium castaneum*.

Among the numerous insect sHSPs genes logged into NCBI, those belonging the following representative species were selected for this study: *Tribolium castaneum, Drosophila melanogaster, Bombyx mori, Apis mellifera, Locusta migratoria*, and *Colaphellus bowringi*. Multiple protein alignments showed that *H. axyridis* sHSPs were highly homologous to that known and predicted for other insects, with HaraxHsp10.87 being 99.0% identical to TricaHsp10 (XP975179.2). Similar molecular weight sHSPs belonging to different insect species appeared in the same branch of the phylogenetic tree (Figure 2).

### Developmental expression patterns of sHSPs

**Figure 3:**
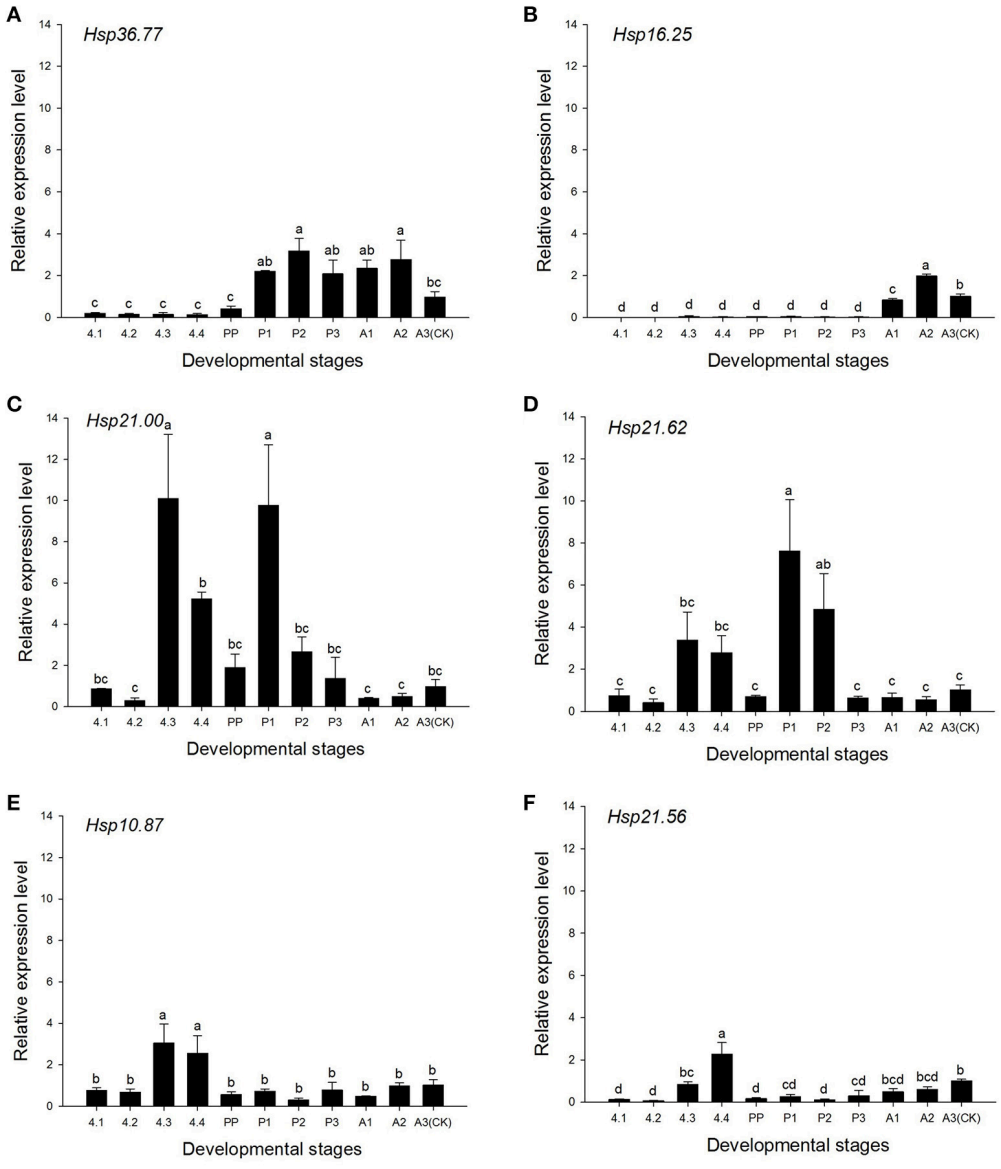
**Relative expression levels of several sHSPs in ***Harmonia axyridis*** in different development stages**. Experimental population is used for this trial. **(A)** *Hsp36.77*. **(B)** *Hsp16.25*. **(C)** *Hsp21.00*. **(D)** *Hsp21.62*. **(E)** *Hsp10.87*. **(F)** *Hsp21.56*.Changes in *H. axyridis* sHSPs mRNA levels from day 1 of fourth-instar larvae to adult stage in relation to those of *Harp49* (*H. axyridis* ribosomal protein 49 gene) were measured by quantitative real-time PCR. 4, fourth-instar larva; PP, pre-pupae; P, pupae; and A, adult. Each combination of letters and numbers represents the age of the individual at a certain developmental stage (e.g., 4.1: the 1st day of fourth-instar larva). Data are presented as means ± s.d. (*n* = 3). Bars with different letters indicate significant differences (*P* < 0.05) and used Tukey's test, α = 0.05, a > b > c.

According to the patterns of the six sHSPs genes determined by qRT-PCR, *Hsp36.7* was continuously expressed from pupal stages to 3-day adults in the *H. axyridis* experimental population [*F*_(10, 25)_ = 23.544, *P* < 0.001; Figure 3A]. The expression of *Hsp16.25* was high in adults but low in larvae and pupae [*F*_(10, 29)_ = 391.653, *P* < 0.001; Figure 3B], and *Hsp21.00* [*F*_(10, 24)_ = 18.652, *P* < 0.001] and *Hsp21.62* [*F*_(10, 26)_ = 13.078, *P* < 0.001] were expressed at higher levels in the later period of fourth-instar larvae and in early pupae than in 3-day adults (Figures 3C,D). A similar trend was found for the expression of *Hsp10.87* [*F*_(10, 28)_ = 14.906, *P* < 0.001] and *Hsp21.56* [*F*_(10, 26)_ = 29.573, *P* < 0.001], which were highly expressed in the later period of fourth-instar larvae (Figures 3E,F).

### Expression of sHSPs under short-term cooling and heating

**Figure 4:**
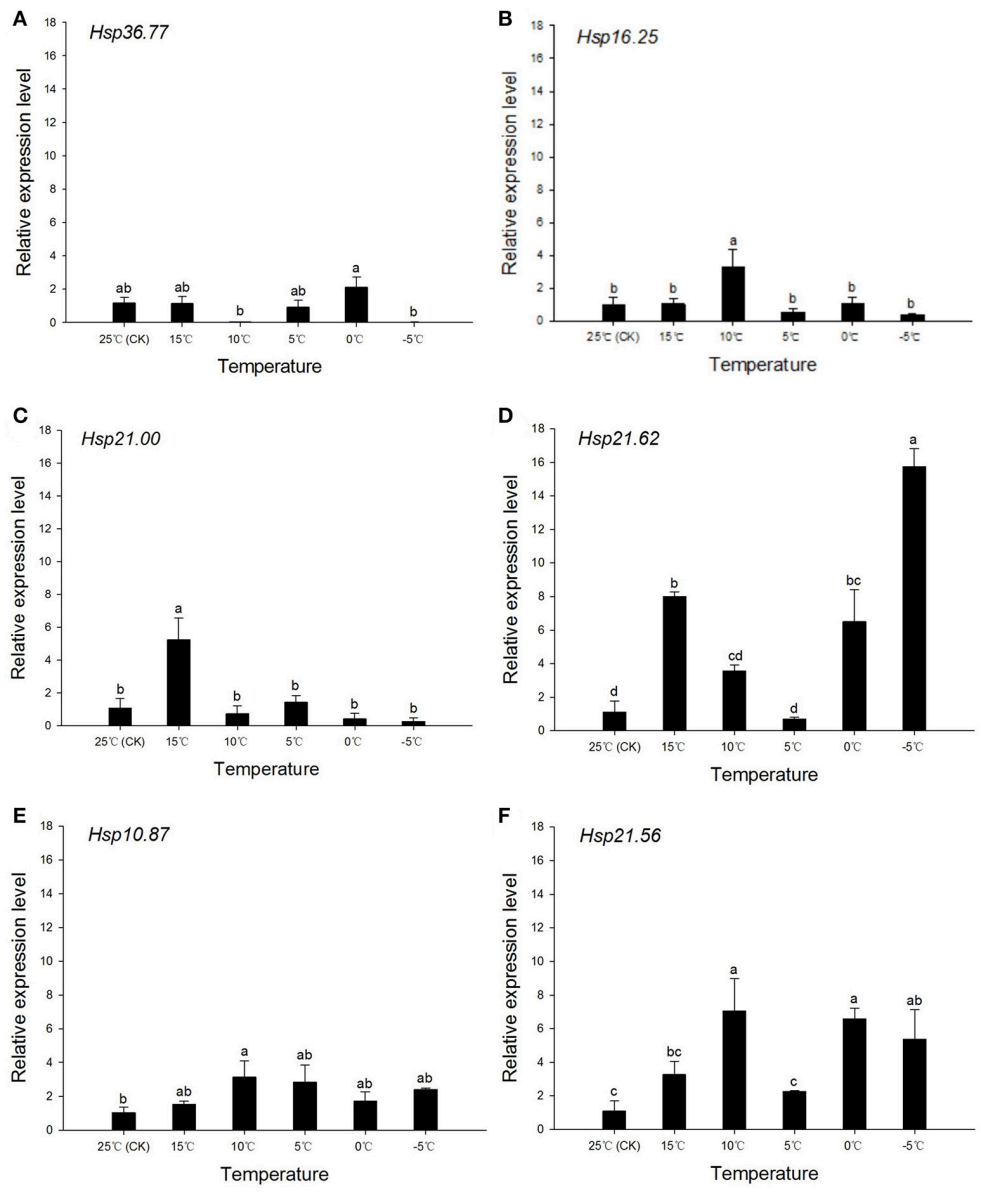
**Relative expression of ***Harmonia axyridis*** sHSPs under the short-term cooling treatment**. Experimental population is used for this trial. **(A)** *Hsp36.77*. **(B)** *Hsp16.25*. **(C)** *Hsp21.00*. **(D)** *Hsp21.62*. **(E)** *Hsp10.87*. **(F)** *Hsp21.56*. Expression of sHSPs was examined in a gradually cooled environment, from 25 to −5°C.During the cooling process, the control group (CK) comprised adults reared at an optimum temperature (25°C) without any cold stimulation. Data are presented as means ± s.d. (*n* = 3). Bars with different letters indicate significant differences (*P* < 0.05) and used Tukey's test, α = 0.05, a > b > c > d.

The mRNA levels of *Hsp36.77, Hsp16.25, Hsp21.00, Hsp21.62, Hsp10.87*, and *Hsp21.56* determined during cooling and heating conditions in the *H. axyridis* experimental populations, revealed complex patterns under cooling conditions. In the short-term cooling treatment, the expression at 25°C was used as reference. The expression of *Hsp36.77* was highest at 0°C and significantly higher than that registered at −5 and 10°C [*F*_(5, 12)_ = 5.421, *P* = 0.023; Figure 4A]; however, it was not significantly different from that observed in the control group (CK). The expressions of *Hsp16.25* [*F*_(5, 11)_ = 7.658, *P* = 0.014] and *Hsp21.00* [*F*_(5, 13)_ = 20.905, *P* < 0.001] were significantly higher at 10 and 15°C, respectively, than at other temperatures (Figures 4B,C). The expression of *Hsp21.62 first* increased when temperature dropped from 25 to 15°C, decreased in the following cooling to 5°C, and reached its highest level at −5°C [*F*_(5, 11)_ = 69.016, *P* < 0.001; Figure 4D]; at this temperature, expression was significantly higher than at any other temperature. The expression of *Hsp10.87* was highest at 10°C [*F*_(5, 13)_ = 4.032, *P* = 0.040; Figure 4E], although it was only significantly different from that obtained at 25°C. Expression of *Hsp21.56* was significantly higher than that of the CK group at 10, 0, and −5°C [*F*_(5, 12)_ = 7.441, *P* = 0.010; Figure 4F].

**Figure 5:**
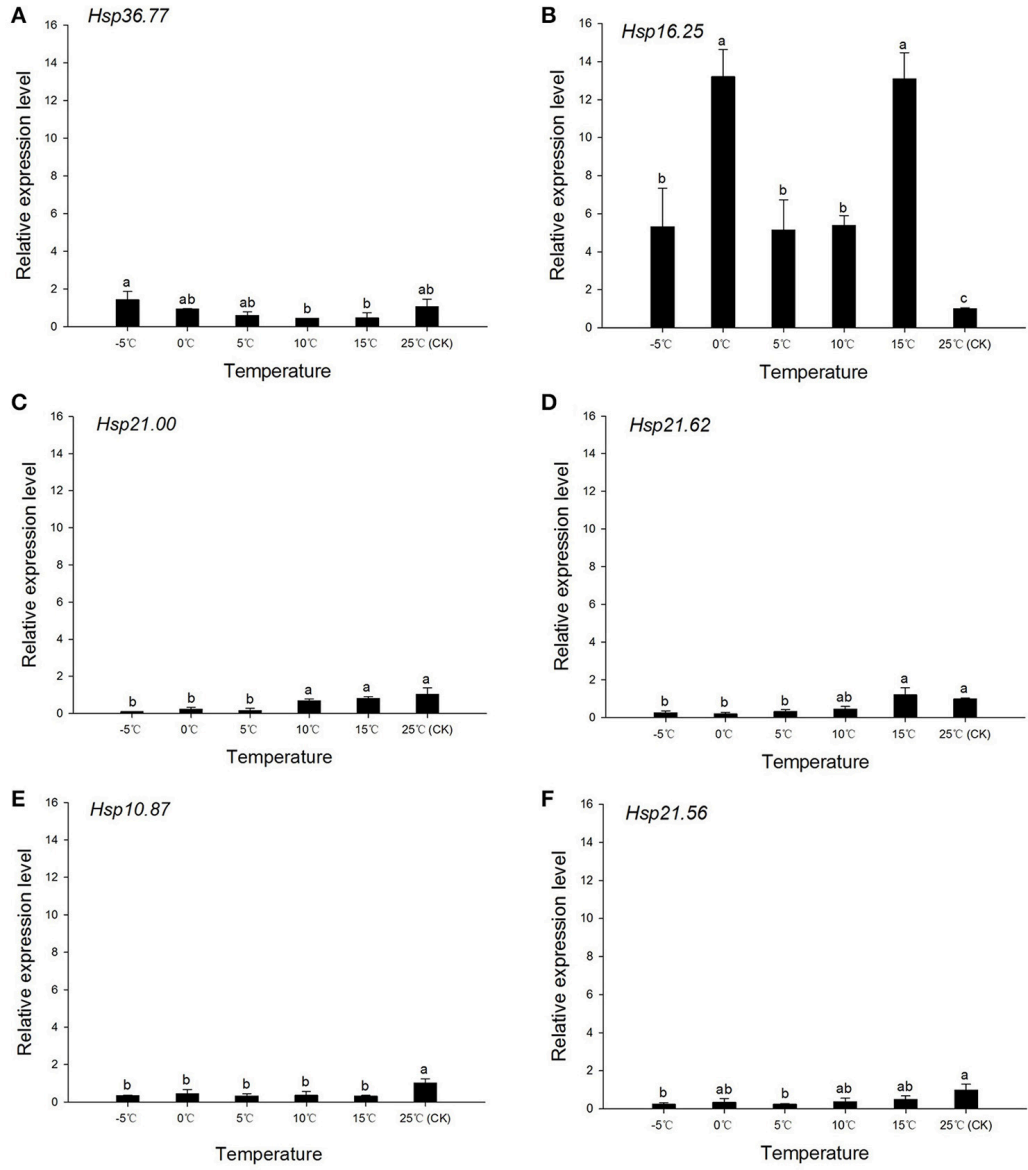
**Relative expression of ***Harmonia axyridis*** sHSPs under the short-heating treatment**. Experimental population is used for this trial. **(A)** *Hsp36.77*. **(B)** *Hsp16.25*. **(C)** *Hsp21.00*. **(D)** *Hsp21.62*. **(E)** *Hsp10.87*. **(F)** *Hsp21.56*. Expression of sHSPs was examined in a gradually heated environment, from −5 to 25°C. During the heating process, the control group (CK) comprised adults reared at an optimum temperature (25°C) without any heat stimulation. Data are presented as means ± s.d. (*n* = 3). Bars with different letters indicate significant differences (*P* < 0.05) and used Tukey's test, α = 0.05, a > b > c.

In the short-term heating treatment, the expression of *Hsp36.77* decreased when temperature changed from −5 to 10°C, increasing afterwards [*F*_(5, 13)_ = 5.946, *P* = 0.014; Figure 5A]; however, expression levels were not significantly different from those registered in the CK. The expression of *Hsp16.25* was significantly higher than that of the CK, especially at 0 and 15°C [*F*_(5, 12)_ = 42.868, *P* < 0.001; Figure 5B]. The expression levels of *Hsp21.00* [*F*_(5, 12)_ = 61.788, *P* < 0.001] and *Hsp21.62* [*F*_(5, 12)_ = 10.262, *P* = 0.004] generally increased with increasing temperature and significantly differed between temperatures below or above 10°C (Figures 5C,D). There was no significant difference in the expressions of *Hsp10.87* and *Hsp21.56* [*F*_(5, 14)_ = 2.438, *P* = 0.116] from −5 to 15°C during the heating process, but the expression level of these genes at 25°C the expression level *Hsp10.87* [*F*_(5, 13)_ = 14.561, *P* = 0.001] was significantly higher than that obtained for the CK (Figures 5E,F).

### Expression of sHSPs in yellow and black forms during low temperature storage

**Figure 6:**
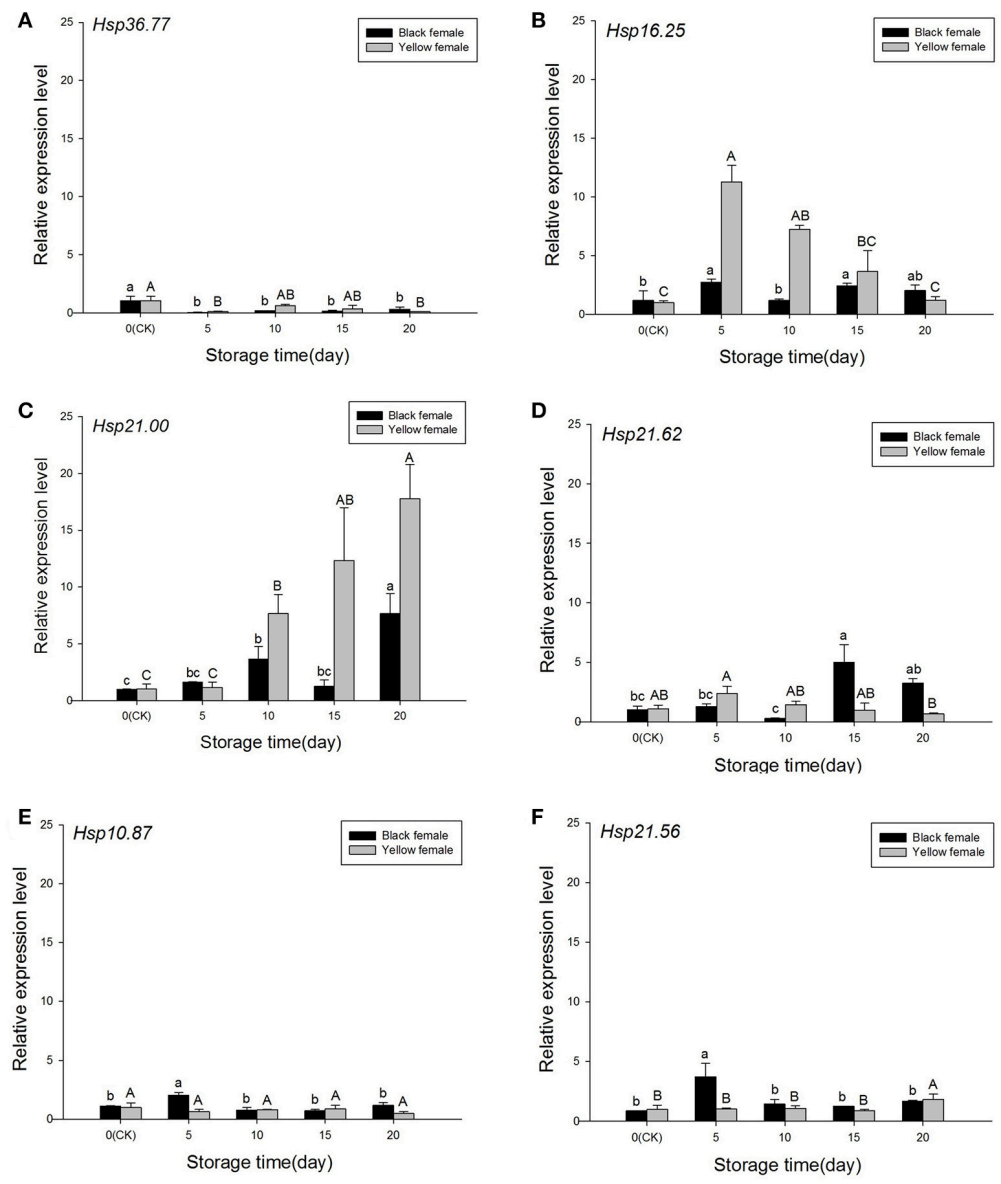
**Relative expression of sHSPs in the experimental population of ***Harmonia axyridis*** under the low-temperature storage treatment**. **(A)** *Hsp36.77*. **(B)** *Hsp16.25*. **(C)** *Hsp21.00*. **(D)** *Hsp21.62*. **(E)** *Hsp10.87*. **(F)** *Hsp21.56*. Selected black and yellow females of the experimental population stored in the refrigerator (5°C) were remove at days 0, 5, 10, 15, and 20. Data are presented as means ± s.d. (*n* = 3). Bars with different letters indicate significant differences (*P* < 0.05) and used Tukey's test, α = 0.05, a > b > c or A > B > C.

During storage at low temperature, the expression levels of sHSPs recorded at day 0 were considered the CK. The expression of *Hsp36.77* was always lower in black [*F*_(4, 12)_ = 10.562, *P* = 0.030] and yellow females [*F*_(4, 9)_ = 6.933, *P* = 0.028] than in the CK (Figure 6A). Compared to the CK, the expression of *Hsp16.25* in yellow females was significantly higher at days 5 and 10 [*F*_(4, 9)_ = 35.738, *P* = 0.001], whereas in black females it was significantly higher at days 5 and 15 [*F*_(4, 12)_ = 5.170, *P* = 0.024; Figure 6B]. The expression of *Hsp21.00* in yellow females was significantly higher than that of the CK at days 10–20 [*F*_(4, 9)_ = 4.147, *P* = 0.075], and it increased with increasing exposure to low temperature; expression levels of *Hsp21.00* in black females at days 10 and 20 were significantly higher than that of the CK [*F*_(4, 9)_ = 16.895, *P* = 0.004; Figure 6C). The expression level of *Hsp21.62* in yellow females was highest at day 5, but not significantly different from that of the CK [*F*_(4, 9)_ = 4.147, *P* = 0.075], while the expression of this gene in black females was significantly higher than that of the CK group at day 15 [*F*_(4, 9)_ = 16.895, *P* = 0.004; Figure 6D]. The expressions of *Hsp10.87* [black females *F*_(4, 11)_ = 15.007, *P* = 0.002; yellow females *F*_(4, 12)_ = 1.909, *P* = 0.202] and *Hsp21.56* [black females *F*_(4, 11)_ = 11.074, *P* = 0.004; yellow females *F*_(4, 11)_ = 4.934, *P* = 0.033] presented a similar trend, and were generally not significantly different from those in the CK group, except in black females at day 5 (both genes) and in yellow females at day 20 (*Hsp21.56*; Figures 6E,F).

**Figure 7:**
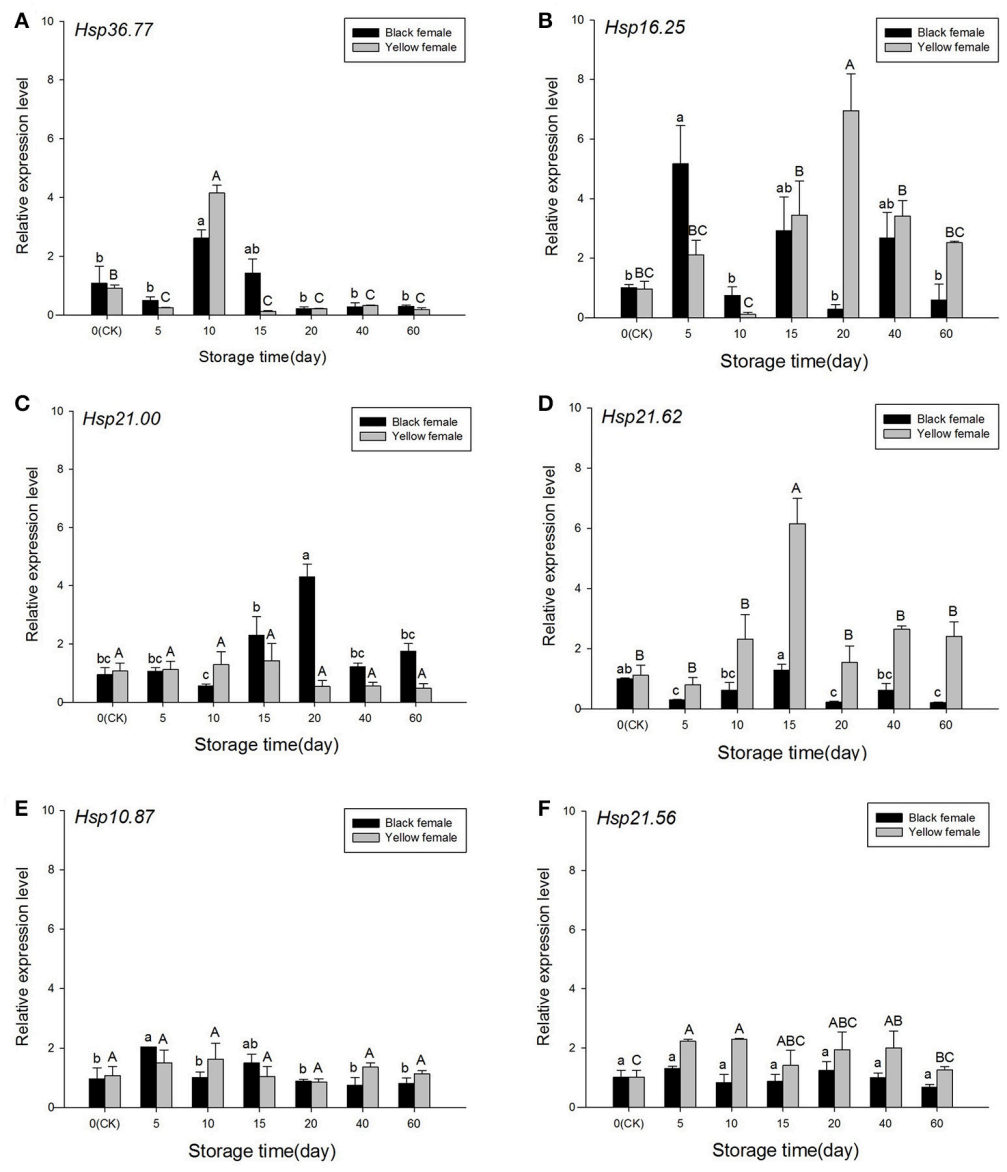
**Relative expression of sHSPs in the overwintering population of ***Harmonia axyridis*** under the low-temperature storage treatment. (A)** *Hsp36.77*. **(B)** *Hsp16.25*. **(C)** *Hsp21.00*. **(D)** *Hsp21.62*. **(E)** *Hsp10.87*. **(F)** *Hsp21.56*. Selected black and yellow females of the overwintering population stored in the refrigerator (5°C) were removed at days 0, 5, 10, 15, and 20. Data are presented as means ± s.d. (*n* = 3). Bars with different letters indicate significant differences (*P* < 0.05) and used Tukey's test, α = 0.05, a > b > c or A > B > C.

Analyses of expression levels in the *H. axyridis* overwintering population revealed *Hsp36.77* was highly expressed at day 10 in both black [*F*_(6, 13)_ = 16.136, *P* = 0.01] and yellow females [*F*_(6, 15)_ = 420.816, *P* < 0.001; Figure 7A], generally differing significantly from expression levels in the control groups. In black females, the expression of *Hsp16.25* was significantly higher than that of the CK group at day 5 [*F*_(6, 13)_ = 10.691, *P* = 0.03], and in yellow females it generally increased from day 0 to day 20 (when it significantly differed from that in CK), decreasing afterwards [*F*_(6, 13)_ = 19.867, *P* < 0.001; Figure 7B]. The highest expression level of *Hsp21.00* in black females found at day 20 significantly differed from that of CK [*F*_(6, 15)_ = 14.069, *P* < 0.001], but there were no significant differences in the expression of this gene in yellow females [*F*_(6, 15)_ = 1.879, *P* = 0.190; Figure 7C]. The expression of *Hsp21.62* was significantly highest at day 15 in yellow females [*F*_(6, 14)_ = 21.062, *P* < 0.001] and black females [*F*_(6, 13)_ = 15.579, *P* = 0.001; Figure 7D], whereas *Hsp10.87* expression was significantly higher at day 5 in black females [*F*_(6, 16)_ = 9.523, *P* = 0.001], but no significant differences were detected in yellow females [*F*_(6, 19)_ = 1.521, *P* = 0.247; Figure 7E]. The expression of *Hsp21.56* in yellow females was significantly higher at days 5, 10, and 40 [*F*_(6, 16)_ = 3.234, *P* = 0.049], whereas no significant differences were detected in black females [*F*_(6, 16)_ = 2.808, *P* = 0.072; Figure 7F].

### Expression of sHSPs in experimental and overwintering populations during low temperature storage

Results showed that the six sHsps have different functions in the regulation of cold hardness in *H. axyridis*. The expression level of *Hsp36.77* decreased in the experimental population (Figure 6A), while it first increased and then decreased in the overwintering population (Figure 7A). Although, *Hsp16.25* expression pattern was similar in experimental and overwintering population, in yellow females the expression of this gene first increased and then decreased while black females showed a different pattern (Figures 6B, 7B). In addition, *Hsp16.25* expression increased with increasing storage time in the experimental population (Figure 6C) and presented an identical trend to *Hsp36.77* and *Hsp21.62* in the overwintering population (Figures 7C,D). The expression of *Hsp21.62* increased in black females from the experimental population and in yellow females from the overwintering population and kept relatively high levels as storage time increased (Figures 6D, 7D). The expression levels of *Hsp10.87* and *Hsp21.56* were stable during storage times and similar between experimental and overwintering population (Figures 6E,F, 7E,F).

## Discussion

Insects are heterothermic organisms, and their survival through winter determines the perpetuation of their populations (Zhang and Ma, 2013). In temperate and cold zones, winter temperature is usually lower than the insects' FP and their overwintering stages generally have a lower SCP than insects living in other zones. This is the case of *L. migratoria* eggs (−26°C), *Liriomyza* spp. pupae (−19°C) (Jing and Kang, 2002), *Chrysoperla sinica* (−13°C) (Guo et al., 2006), *Ectomyelois ceratoniae* diapausing larvae (−17.3°C) (Heydari and Izadi, 2014), and *Pityogenes chalcographus* (−26.3°C) (Koštál et al., 2014). In the present study, *H. axyridis* were divided into black (melanic) and yellow (non-melanic) forms according to the background of their elytra, and into experimental and overwintering populations according to their source. The SCP and FP of the overwintering population was significantly lower than that of the experimental population for the same forms of *H. axyridis* (Figure 1), and females had a lower SCP than males, in either black or yellow *H. axyridis* (Figure 1B). Thus, overwintering populations seem to decrease their SCP to avoid the damages caused by fluid freezing, which agrees with the previously described seasonal changes in *H. axyridis* SCP and its significant decrease in winter (Zhao et al., 2008). Some studies reported that insects with low SCP had high levels of trehalose in the winter (Heydari and Izadi, 2014; Koštál et al., 2014; Vallières et al., 2015). This is an important winter adaptation strategy for survival at low temperature. Although, we found no significant differences in average water content between experimental and overwintering populations (Figure 1A), previous research found seasonal variations in the water contents of male and female *H. axyridis* adults, which decreased with decreasing temperature (Zhao et al., 2008). Water loss before winter has also been reported in other insects (Nedved et al., 1998; Holmstrup et al., 1999).

The sHSPs are HSPs that function as molecular chaperones (Li et al., 2009). Their molecular weight ranges from 12 to 43 kDa and is usually bellow 30 kDa (Kim et al., 1998; Waters et al., 2008). In *H. axyridis*, one *Hsp90* and three *Hsp70* family genes were cloned and reported as highly conserved (Tang et al., 2010; Shen et al., 2015). The homology of *HaHsp90* with other insect *Hsp90*s varied from 81 to 90%, which was the homology with *T. castaneum* (Tang et al., 2010); *HaHsp70* also had a high sequence homology with other eukaryotic *Hsp70s*, the highest also with *T. castaneum* (93%) (Yang et al., 2009; Shen et al., 2015). Insect HSP70s have three signatures -“IDLGTTYSCVGV,” “IFDLGGGTFDVSIL,” and “IVLVGGSTRIPKIQ,” one ATP/GTP binding site motif, “AEAYLG(K/T)T,” and a “MEEVD” motif at the C terminus (Shen et al., 2015). Members of the HSP90 family also have an “MEEVD” motif at the C terminus, but this was not found in sHSPs (Figure S1). These were reported to have a conserved secondary structure and functional domain (Sun and MacRae, 2005), and sHSPs with similar molecular weight were found in the same branch of the phylogenetic tree (Figure 2).

Small HSPs are molecular chaperones not only under stress conditions, but also during normal development (Sun and MacRae, 2005). Under thermal and other damaging stresses, sHSPs bind to other cellular proteins protecting them from denaturation; in addition, they participate in protein folding and transportation, embryo development, and immunization mechanisms (Li et al., 2009). In addition, the overexpression of sHSPs can enhance the tolerance of cells to heat shock and to temperature changes. Under normal or stress conditions, HSP90 is present in the cytoplasm of all cell types, where it acts as a molecular chaperone to recover the folding state of the denatured protein (Yonehara et al., 1996). Studies have shown that sHSPs are widely distributed in insects, and are closely related to their growth and development (Sun and MacRae, 2005). In the present study, *Hsp16.25* was highly expressed in adults and *Hsp36.77* was highly expressed from 1-day pupae to 3-day adults (Figures 3A,B). A previous study reported that *HaHsp68* was highly expressed from 2-day fourth-instar larvae to 3-day adults, while *HaHsp70A* was highly expressed in larvae (Shen et al., 2015). In the present study, *Hsp21.00, Hsp21.62*, and *Hsp10.87* expression levels varied according to developmental stages, whereas *Hsp21.56* expression increased gradually from 2-day pupae to 3-day adults, suggesting *Hsp21.56* and *Hsp16.25* might have important functions in adults (Figure 3). Thus, sHSPs genes might play different roles during *H. axyridis* growth and development.

The expression of *Hsp70, Hsp74*, and *Hsp83* among the several developmental stages of *Spodoptera exigua* differed between fat body and whole body tissues (Xu et al., 2011). *Drosophila* spp. long-term exposure to 0°C induced HSPs expression in the whole body (Burton et al., 1998) and cold-shock treatment induced the expression of *Hsp90, Hsp70, Hsp40*, and some sHSPs in *Liriomyza sativae* (Huang and Kang, 2007). The expression level of *SeHsp70* and *SeHsp74* increased quickly at 40°C, while *SeHsp83* expression increased with heat-shock exposure time. These genes were also differently expressed during cold-shock treatment at 0°C: whereas *SeHsp70* was highly expressed after 15 min of cold-shock, followed by a decrease from min 15 to min 60, *SeHsp74* and *SeHsp83* expressions increased from min 15 to min 45 (Xu et al., 2011). When *H. axyridis* adults were subject to different low temperatures, *Hsp70B* expression at 0°C was higher than that of the control group and higher than at other temperature conditions (Shen et al., 2015). Under the short-term cooling treatment performed in the present study, the expression of *Hsp16.25* increased significantly at 10°C, followed by a decrease (Figure 4B), *Hsp21.00* expression increased significantly at 15°C and then decreased (Figure 4C), and *Hsp10.87* expression was highest at 10°C (Figure 4E); *Hsp21.62* and *Hsp21.56* expressions were highest at 15, 0, and −5°C, and were always higher than that of the control group (25°C; Figures 4D,F). These results indicated that sHSPs might increase their expression to provide insects' with cold-hardiness. Given that, during the short-term heating treatment, *Hsp21.00, Hsp21.62*, and *Hsp21.56* expressions increased gradually from 10 to 25°C (Figures 5C,D,F), and *Hsp10.87* expression was higher than that of the control group (at −5°C; Figure 5E), sHSPs might also play a key role in heat-stress conditions.

Populations of the ladybird *H. axyridis* contain both melanic and non-melanic forms, and changes in allele frequency in some populations suggested melanism might be advantageous in winter but costly in summer (Michie et al., 2010; Wang et al., 2011). The ratio of *H. axyridis* elytral coloring varied seasonally (Wang et al., 2009), which might be related to the protection conferred by color (Geng and Tan, 1980), and is related to temperature changes (Michie et al., 2010; Purse et al., 2015; Roy et al., 2016). Previous research indicated that black and yellow populations of *H. axyridis* might have had different roles in temperature or climatic adaptation (Roy et al., 2016), so we analyzed *sHSPs* expression under different storage times in black and yellow female populations, separately. In the yellow population, *Hsp16.25* and *Hsp21.62* expressions increased significantly at day 5, decreasing gradually afterwards (Figures 6B,D), while *Hsp21.00* and *Hsp10.87* expressions increased gradually (Figures 6C,E), and *Hsp21.56* expression was relatively stable, except at day 20 (Figure 6F). In the black population, *Hsp16.25, Hsp10.87*, and *Hsp21.56* were highly expressed at day 5, decreasing and stabilizing afterwards (Figures 6B,E,F), while *Hsp21.62* expression was higher after day 15 (Figure 6D). In the experimental population, five of the studied genes played a role during cold-temperature storage and their function varied between melanic and non-melanic forms of *H. axyridis* adults. Thus, there might be differences between cold-resistance in *H. axyridis* melanic and non-melanic forms. There are many reasons for the elytral variety observed in *H. axyridis* (Tang et al., 2012), including mating selection, so the differences found in gene expression need to be further studied.

Winter in temperate zones imposes a substantial environmental stress on arthropods (Kang et al., 2009). Many studies revealed that cold acclimation, especially between 0 and 5°C, significantly improves cold tolerance in insects (Broufas and Koveos, 2001). Cold acclimation may induce the accumulation of cryoprotectants (such as trehalose, glycerol, and polyol; Koštál et al., 2001; Slachta et al., 2002), and the synthesis of antifreeze proteins (HSP20.5, HSP70, and HSP90; Wang et al., 2006; Feng et al., 2007). Many insects mitigate seasonal stresses by entering diapause (Dong et al., 2014). Under the low-temperature storage experiment, the overwintering black population revealed a high expression of *Hsp36.77* at day 10 and of *Hsp16.26* and *Hsp10.87* at day 5 followed by a decrease (Figures 7A,B,E), while *Hsp21.00* presented a relatively high and stable expression from day 15 to day 60 (Figure 7C). In the yellow population, *Hsp21.56* was highly expressed during the 60 days of cold storage (Figure 7F), *Hsp36.77* was highly expressed at day 10, *Hsp16.25* at day 20, and *Hsp21.62* at day 15, all followed by a decrease (Figures 7A,B,D). The high expression of these genes might promote the expression of other cold-resistant genes and protect *H. axyridis*, as sHSPs seem to participate in stress response under low-temperature storage. As the cold winter starts, overwintering populations are ready to survive it, because they have adapted their physiology and behavior by entering diapause, decreasing body water content and SCP, accumulating fat, finding hiding places, etc. (Zhao et al., 2008). Moreover, overwintering populations live longer than experimental populations at appropriate storage temperatures (Ruan et al., 2012).

In summary, the present study found that average water content was not significantly different between adults of both populations, but the SCP and FP of the overwintering population were significantly lower than that of the experimental population. Differences in sHSPs expression between experimental and overwintering populations were correlated with *H. axyridis* elytral coloring, cooling, and heating and low temperature storage, suggesting that the mechanism of cold resistance might differ among black and yellow females of *H. axyridis*. In addition, different sHsps might play different roles in the cold hardness process of *H. axyridis* populations.

## Ethics statement

All applicable international, national, and/or institutional guidelines for the care and use of animals were followed.

## Author contributions

Conceived and designed the experiments: SW, ZM, S-GW, BW, and BT. Performed the experiments and analyzed the data: HW, ZS, QS, and CX. Contributed reagents/materials/analysis tools: SW, ZM, S-GW, and BT. Wrote the paper: HW, SW, and BT.

### Conflict of interest statement

The authors declare that the research was conducted in the absence of any commercial or financial relationships that could be construed as a potential conflict of interest.

## Supplementary material

Figure S1 **The cDNA sequence of ***Harmonia axyridis*** sHSPs**. The initiation and termination codons are indicated in bold and underlined. **(A)** *Hsp36.77*, **(B)** *Hsp16.25*, **(C)** *Hsp21.00*, **(D)** *Hsp21.62*, **(E)** *Hsp10.87*, **(F)** *Hsp21.56*.
