# Supplementary material for: Molecular Cloning and Induced Expression of Six Small Heat Shock Proteins Mediating Cold-Hardiness in Harmonia axyridis (Coleoptera: Coccinellidae)
Source: Front Physiol. 2017 Feb 9;8:60. doi: 10.3389/fphys.2017.00060 (PMC5299025; doi:10.3389/fphys.2017.00060)
Supplement: Figure S1 — The cDNA sequence of Harmonia axyridis sHSPs. The initiation and termination codons are indicated in bold and underlined. (A) Hsp36.77, (B) Hsp16.25, (C) Hsp21.00, (D) Hsp21.62, (E) Hsp10.87, (F) Hsp21.56. [file Image1.PDF]

Supplementary Figure 1

A

1 **ATG**GGTAAAGACTACTACTCCATATTAGGTATACGTAAAAACTCGACAGATGACGAAATAAAGAAAGCCTACCGAAAATTAGCCTTACAA.

M G K D Y Y S I L G I R K N S T D D E I K K A Y R K L A L Q.

91 TATCATCCCGACAAAAACAAATCCCCCAAAGCCGAGGAGAAATTCAAAGAAATCGCAGAAGCTTACGAGGTTCTATCCGACAAGAAGAAA.

Y H P D K N K S P K A E E K F K E I A E A Y E V L S D K K K.

181 CGAGACATTTACGACAATTACGGTGAAGACGGTTTGAAGGGGGGAGCAGGCCCTCATGGAAACACAGGATTCACTTACACATTCCACGGG.

R D I Y D N Y G E D G L K G G A G P H G N T G F T Y T F H G.

271 GATCCGAGGGCAACTTTCGCTCAGTTCTTCGGCAATACGAATCCATTTCAGAGCTTTTTCGGTTTCGACGATTCCTTCGACAACGACATG.

D P R A T F A Q F F G N T N P F Q S F F G F D D S F D N D M.

361 AACCAATCGACCTTCATCTTCGGATTTCCAGGTGCAGAAAATCACGTGGGATCACACCGGGAAAGAATGGCCCAAGATCCACCGGTGGAG.

N Q S T F I F G F P G A E N H V G S H R E R M A Q D P P V E.

451 CACGAAATTTATCTAACTTTGGAGGAGGTTCTTCACGGCTGTCTTAAAAAGATGAAAATACAGAAGAAAATCCTGCTTCCTAATGGCAAG.

H E I Y L T L E E V L H G C L K K M K I Q K K I L L P N G K.

541 TTGGGAAAGGAGGAAAAGATGCTGGAAATAAATGTTCAACCTGGTTGGAAAGCCGGTACTAAGATAACATTTCCCAAAGAAGGGGACCAG.

L G K E E K M L E I N V Q P G W K A G T K I T F P K E G D Q.

631 GGGGTCAACAAGATACCTGCAGATATAGTTTTCATTATAAGAGATAAAACACACAGTACCTTCAAGAGGGAAGGCAGTGATTTGAGATAC.

G V N K I P A D I V F I I R D K T H S T F K R E G S D L R Y.

721 ACAGCTAAGATTTCCTTAAAACAAGCATTATGTGGATGTATAGTAAATATTCCTCTTATTGGTGGAGGTCAGGAAGCTCTTAACTATAAA.

T A K I S L K Q A L C G C I V N I P L I G G G Q E A L N Y K.

811 AATGATATCATAAAACCTCAAACTATCAGACGTATTGCAGGTAGAGGTCTACCTTTCCCGAAAGAACCTTCCAGAAGAGGAGATCTAATA.

N D I I K P Q T I R R I A G R G L P F P K E P S R R G D L I.

901 GTAACATTTGATATTCGGTTTCCCGAAAGTCTACCAACAGCTACGCGAAAGTATTTAGAAGAAAAATTACCAGCT**TGA**.

V T F D I R F P E S L P T A T R K Y L E E K L P A *.

B

1 **ATG**CCGAAACTTTTCAACAAAAAAGAATCTCCGAAAACATCAAAATCCACGGATACTAAAGAAGAACCTTCAGATGAACACGATTTTATT.

M P K L F N K K E S P K T S K S T D T K E E P S D E H D F I.

91 CGAAAAGAATTTGGAGATTACTGGAAGAACCCAAAGAACGACAAGTTCGAGAGGATCCTCAAAGATGGAGACACCAAACCGAAGAAGAAA.

R K E F G D Y W K N P K N D K F E R I L K D G D T K P K K K.

181 GACTTCCAGTTGACCCTCAACATACCAGAGTACAAACCGGAAGAGGTGTTGGTAGCAGCCAACGGAAATGTGGTGGAAGTAAAGGGTAAG.

D F Q L T L N I P E Y K P E E V L V A A N G N V V E V K G K.

271 CATCAAGAAAGGGACGAAAAAGGTGAAATGCAGACTGTTAGAAGTTTTATCAAGTCCTTCAGTATATCAGAAGATTGTGATGTATCCCAG.

H Q E R D E K G E M Q T V R S F I K S F S I S E D C D V S Q.

361 CTGAGGAGTAAGTTTGAGAAGGAGGGTGTCCTCACCATCAGTGCACCGAGGAAGACTGAA**TAG**.

L R S K F E K E G V L T I S A P R K T E *.

C

1 **ATG**TCTCTTTTACCGTTCCTGCTAGACAGTTACGTTCGTCCTACCCGACTTTTGGACCAGCAGTTTGGCTTGTGTCTTGATCCCGAAGAC.

M S L L P F L L D S Y V R P T R L L D Q Q F G L C L D P E D.

91 CTTTTTGCACCGGTGGTTGATCCCCGACAAATGCTGGGTCAATTCAGGGGTCCTGTGGGATACATACGTCCTTGGAAGTCCGCATTATCT.

L F A P V V D P R Q M L G Q F R G P V G Y I R P W K S A L S.

181 CAAAAAGACGTCGGATCAACTCTGGCTATCGACAAGGACAGTTTCAAAGCCAACTTGGACGTGCAACAGTTCAGACCAGAAGAAATAACT.

Q K D V G S T L A I D K D S F K A N L D V Q Q F R P E E I T.

271 GTCAAATCCAGTGGAAACAACACCATCACCATAGAAGGCAAGCATGAGGAAAAACAGGACGAGCATGGATTCATCTCCAGGCAATTTGTA.

V K S S G N N T I T I E G K H E E K Q D E H G F I S R Q F V.

361 AGAAAATACGTCCTTCCTAAAGACTGCGATATCGAAAAGGTGGAATCGAGATTGTCTTCGGATGGCGTGCTTACCATAATCGCACCAAGG.

R K Y V L P K D C D I E K V E S R L S S D G V L T I I A P R.

451 AACAAACCTGAGGAATTGGAAAACAAGACCATTCCCATAACTCAAACAGGACAACCGGCCAAGATTGAACAGAAGAAAGAAGAGGAAGAG.

N K P E E L E N K T I P I T Q T G Q P A K I E Q K K E E E E.

541 AAAGAGGAGAAG**TGA**.

K E E K *.

D

1 **ATG**TCTCTTCTACCGTACATTTTGGATGCCTACACTAGGCCCTCCCGTCTTCTGGACCAACATTTCGGCCTGTCTTTGGACTCCGACGAT.
M S L L P Y I L D A Y T R P S R L L D Q H F G L S L D S D D.

91 CTTCTGAGCCCCATCACCGATCCCAGCATTCTCGCAACATGTCTGAGGTCCCATCCAACCATGGGTTACATCCGTCCCTGGAGAACATCA.
L L S P I T D P S I L A T C L R S H P T M G Y I R P W R T S.

181 GTGTCCCAAAGAGACGTTGGATCCACCATCTCCATGGATAAGGAGAGGTTCCAAGCCAATTTGGATGTTCAACAGTTCAAACCGGAGGAA.
V S Q R D V G S T I S M D K E R F Q A N L D V Q Q F K P E E.

271 ATCACCGTTAAAGCCACTGGAAACAACGTCATTACCATCGAGGGAAAACATGAAGAGAAACAGGATGATCATGGTTATATTTCCAGGCAG.
I T V K A T G N N V I T I E G K H E E K Q D D H G Y I S R Q.

361 TTTGTTAGGAAATACGTAATTCCTAAAGAATGCGATTACGAGAAGATCGAGTCGAAATTGTCTTCTGATGGTGTTCTTACCATCACGGCC.
F V R K Y V I P K E C D Y E K I E S K L S S D G V L T I T A.

451 CCAAGAATCAACGTGGATAAGAGCATTGATCATAAAACCATCCCGATAATCAAGACCGGCCATCCTGCTAAGGTTGAACAGAAAAAGGTC.
P R I N V D K S I D H K T I P I I K T G H P A K V E Q K K V.

541 GAAGAGAAGAAGCCTGAAAATGCAGAGAAG**TAA**.
E E K K P E N A E K *.

E

1 **ATG**TCTGGAGCAGTAAAGAGATTATTGCCCTTGTTGGATAGAGTTTTGGTCAAAAAAGCTGATGCTATCACTACAACAAAAGGAGGAATT.
M S G A V K R L L P L L D R V L V K K A D A I T T T K G G I.

91 GTTATTCCTGACAAAGCTGCAGCAAAAGTCCTGAAGGCAACAGTTGTAGCCGTAGGGCCTGGAGCAAGAAATAAAAATGGAGATCACATT.
V I P D K A A A K V L K A T V V A V G P G A R N K N G D H I.

181 CCCCTTGCTGTCAAAGTTGGGGATAACGTATTACTTCCAGAATATGGTGGAACTAAAGTTGAAATCGAAGAGAATAGCGAATACCATCTA.
P L A V K V G D N V L L P E Y G G T K V E I E E N S E Y H L.

271 TTCAGAGAATCTGACATCCTAGCTAAAATAGAAAAT**TAA**.
F R E S D I L A K I E N *.

F

1 **ATG**CCGAAAAGCGCCGAGAAAAGCAGACCCTGCCACATCCCAGATTCCCAATTCGGCATTTGCTATGATCCTGAACAGCTCCTGAGAAAC.
M P K S A E K S R P C H I P D S Q F G I C Y D P E Q L L R N.

91 ATCCATCTTAACGAGCTCGAAAGTCCTACAGAGCTCATAGAGCTTCCTCACGGTTACATCAGGCCTTGGAAAGACCCAACCCAACATGAT.
I H L N E L E S P T E L I E L P H G Y I R P W K D P T Q H D.

181 GCCGGCTCTACAGTTTTTATGGACAAACACAAGTATAAGGCCATGCTGGATGTTTTCCAGTTCAAGCCTGAAGAACTTAAGGTCACCATC.
A G S T V F M D K H K Y K A M L D V F Q F K P E E L K V T I.

271 GAAGGAGTACATATGGTGGCCGTCGAGGGAAATCAGGAGAAAAAGGAGGAGCATGGTGGACTGGTAGATCGCTATTTCAAGAGGAAATAT.
E G V H M V A V E G N Q E K K E E H G G L V D R Y F K R K Y.

361 CTGGTACCACCATTTTACGATTGCCAGAAAGTGAAGTGCAAAATGTCAGCTGATCGGATTATCGTGATCACCATTCCGAGACTAGATCCC.
L V P P F Y D C Q K V K C K M S A D R I I V I T I P R L D P.

451 AAACTAACAGAGGAAAAAATCGAAGTTCCTCTCGTTCAAACTGAACAAAACATTAGAGATGAAGTTAAGGAGTTGCTGGAGAAAATTAAG.
K L T E E K I E V P L V Q T E Q N I R D E V K E L L E K I K.

541 GAAGACAATTAT**TGA**.
E D N Y *.
